# Coleções coloniais em Portugal e suas invisibilidades: João Jardim e o Museu Municipal da Figueira da Foz

**DOI:** 10.1590/S0104-59702025000100031

**Published:** 2025-09-08

**Authors:** Maria Figueira, Quintino Lopes, Elisabete Pereira

**Affiliations:** i Projeto TRANSMAT/Museu Municipal Santos Rocha. Figueira da Foz – Portugal; ii IN2PAST/Instituto de História Contemporânea/Universidade de Évora. Évora – Portugal figueimaria@gmail.com; iii IN2PAST/Instituto de História Contemporânea/Universidade de Évora. Évora – Portugal qmjl@uevora.pt; iv IN2PAST/Instituto de História Contemporânea/Universidade de Évora. Évora – Portugal ejsp@uevora.pt

**Keywords:** Coleções coloniais, Campanhas militares, Seções de etnografia, Pesquisa de proveniência, João dos Santos Pereira Jardim (1865-1907, Colonial collections, Military campaigns, Ethnography sections, Provenance research, João dos Santos Pereira Jardim (1865-1907

## Abstract

Conhecer os atores associados ao desenvolvimento das coleções contribui para rastrear os traços de colonialidade do discurso dos museus. O caso de estudo de João dos Santos Pereira Jardim, principal doador de uma coleção da seção de etnografia do Museu Municipal da Figueira da Foz, confirma que as campanhas militares nos territórios coloniais são momentos vitais de enriquecimento das coleções dos museus portugueses. Reconstituindo o percurso e o significado de dois objetos recolhidos por esse militar evidenciamos como essa tipologia de atores contribuiu para o crescimento das coleções e para as prevalecentes narrativas eurocêntricas dos museus.

## Abstract (en)

Learning about the actors associated with the development of collections helps trace the signs of coloniality in museum discourse. The case of João dos Santos Pereira Jardim, the main donor of a collection in the ethnography section of the Municipal Museum of Figueira da Foz, confirms that military campaigns in colonial territories were key to enriching the collections of Portuguese museums. By reconstructing the journey of this military officer and the significance of two objects he collected, we reveal how this type of actor contributed to the growth of collections and prevailing Eurocentric narratives in museums.

A história das coleções coloniais dos museus portugueses é um um tema que tem despoletado um crescente interesse académico nos últimos anos. Apesar disso, pouca atenção tem sido conferida ao papel dos agentes coloniais na construção dessas coleções e à sua influência nos processos de produção de conhecimento que perduraram nas narrativas museológicas até à atualidade. Os estudos disponíveis tendem a associar a relação das instituições portuguesas com o colonialismo, como mostram Inês Ponte (2022) e Márcia Chuva (2020) no caso do Museu Nacional de Etnologia.^1^

O museu etnográfico é, à imagem do que diversos autores defendem, um arquivo colonial, pelo que promovendo investigações sobre a história das coleções e dos agentes envolvidos na sua construção nos alerta para a predominância das suas narrativas nos discursos dos museus. A história da ciência tem refletido sobre os processos por meio dos quais a história dos museus se revela na história da construção de coleções, nomeadamente nos processos de atribuição de significados aos objetos (Alberti, 2005). Nesse contexto, tem-se analisado o papel preponderante das redes de atores secundários na construção das coleções dos museus, nomeadamente nos museus de arqueologia portugueses (Pereira, 2017). Essa abordagem mostra como os objetos dos museus nos permitem aceder aos contextos históricos e sociais que envolvem a construção de coleções e produção de conhecimento e que são frequentemente invisibilizados nas narrativas institucionais (Pereira, Nunes, 2019).

A pesquisa de proveniência tem sido aplicada como metodologia de estudo das coleções coloniais em museus por toda a Europa. Os resultados têm promovido novas narrativas (Deußen, 2022, p.2) e evidenciado as conexões das instituições com a execução do poder imperial, sinalizando como as coleções resultam frequentemente da pilhagem de bens culturais (Hicks, 2020).^2^

O estudo da incorporação de objetos de uma seção de etnografia do Museu Municipal Santos Rocha, na Figueira da Foz (Portugal), um museu de arqueologia criado no século XIX, mostrou como uma coleção foi formada no decorrer de comissões e campanhas comandadas pelo militar João dos Santos Pereira Jardim (1865-1907) durante o seu período de serviço nos territórios coloniais. Recorrendo à matriz criada pelo projeto TRANSMAT^3^ traçamos a “biografia das redes” (Foster, 2012) e desenvolvemos a pesquisa de proveniência (Grimme, 2020; Van Beurden, Gondola, Lacaille, 2023; Von Oswald, 2022), que nos permitiu a reconstituição do complexo itinerário de dois objetos dessa coleção. O artigo começa por apresentar uma biografia de João Jardim, evidenciando como a sua relação com o Museu Municipal da Figueira da Foz é apoiada pela sua atuação nos territórios coloniais entre o final do século XIX e o início do século XX. Analisa-se a importância dessa realidade na formação das coleções de Timor-Leste e Angola que foram por si doadas ao museu. Reconstituimos o percurso dessas coleções no museu e os significados que assumiram ao longo do tempo. Por meio da análise de textos produzidos pelo militar e de vários catálogos e roteiros em que constam registos dos objetos por si doados ao museu, analisamos como foi interpretado e produzido o conhecimento, destacando-se o caso de estudo do “Sasando” proveniente de Timor-Leste (MMSR, 2024b) e do “Deus da Guerra”/“Nkisi” proveniente de Angola (MMSR, 2024a). O artigo reflete sobre o impacto que a reconstituição da biografia dos objetos pode assumir na história das coleções coloniais dos museus, contribuindo para a revisão das narrativas museológicas eurocêntricas que prevalecem na instituição.

## Documentar coleções coloniais nos museus

A identificação das doações e do importante papel que os doadores assumem na história dos museus e do colecionismo tem sido destacado por vários autores (Alberti, 2005; Byrne et al., 2011; Dudley et al., 2012). Os objetos doados materializam relações que se desenvolvem entre várias pessoas, não só os seus coletores como outros agentes que fizeram parte do seu percurso, nomeadamente atores relacionados com processos de comercialização (Bouchard, 2023, p.154). O levantamento dos dados biográficos desses atores que participaram na construção de coleções promove a sinalização dos contextos de recolha e outros momentos da história dos objetos, ajudando a esclarecer os significados que assumem, antes e depois de entrarem nos museus (Byrne et al., 2011, p.4). Integrados em complexas redes de conexões, processos de significação, uso, consumo e mercantilização, do passado ao presente, os objetos convocam uma série de agentes que imprimem neles as suas vivências pessoais, economias morais e visões do mundo (Alberti, 2005; Appadurai, 1986).

Em “A bundle of relations: collections, collecting and communities”, Joshua Bell (2017) afirma que o conceito de “biografias culturais dos objetos” desenvolvido por Kopytoff (1986) foi mais tarde elaborado nas ideias de “itinerário” (Joyce, Gillespie, 2015) e “redes biográficas” (Foster, 2012). Fazendo uso da “teoria das redes” de Latour (2005), esses autores focam as formas como as relações dos objetos se constroem e como o seu mapeamento contribui para compreendermos como são fontes de conhecimento indissociáveis dos contextos que produzem os seus significados ao longo da sua vida (Bell, 2017, p.246).

A designação de Foster (2012) apela às relações mais amplas dos objetos. Já a de Joyce e Gillespie (2015) chama a atenção para a dificuldade em limitar as fases de vida dos objetos, propondo romper com a ideia de progresso linear que o conceito de biografia encerra (Bell, 2017, p.246).

Nos museus, as coleções coloniais representam construções de significados fruto das relações produzidas pelos colecionadores e pelos próprios museus (Bachmann, Berazategui, 2022, p.21). Essas coleções reproduzem as narrativas de assimetria entre aquele que é exposto e aquele que expõe, que marcam os discursos coloniais presentes na história dos museus ocidentais (Bennett, 2005; Grimme, 2020). Em parte, essa realidade devese ao processo de construção das coleções, marcado pelo fato de as recolhas de objetos frequentemente ocultarem as relações sociais nas quais se inserem e pela sua documentação ser realizada parcialmente e/ou mediante a perspetiva do coletor (Bell, 2017, p.245). Nesse procedimento, as perspetivas das populações de origem dos objetos têm sido parciais ou totalmente excluídas dos processos de conhecimento e formação de coleções etnográficas dos museus, sendo frequentemente alienadas das narrativas expositivas (p.246). As coleções coloniais apresentam-se, assim, como espaços de negociação em que são geridas formas de ver o mundo e princípios de conhecimento que podem ser contextualizados em lugares e temporalidades particulares, mas que não são visíveis nas narrativas dos museus. No caso apresentado, uma coleção de 260 objetos reporta momentos da história do colonialismo português, especificamente uma comissão de serviço da marinha portuguesa em Timor-Leste e uma campanha militar em Angola, entre o final do século XIX e o início do século XX.

## João Jardim, a coleção de Timor-Leste e o caso do “Sasando”

A investigação realizada no âmbito do projeto TRANSMAT revelou João dos Santos Pereira Jardim como o principal doador da coleção colonial do Museu Municipal Santos Rocha. Oficial da armada, realizou várias comissões militares nas ex-colónias portuguesas. Entre 1898 e 1899, assumiu funções a bordo de vários navios que circulavam entre Macau e Timor-Leste (Portugal, 1885-1906). Poucos anos depois, em 1902, desempenhou o cargo de “governador do distrito do Congo” (Portugal, 1885-1906), um posto administrativo no norte de Angola, onde assumiu o comando da “Expedição à Quincunguila, na circunscrição do Ambrizete” (Jardim, 1903a, p.252). Entre 1904 e 1907 esteve ao serviço da Companhia de Moçambique, onde acabou por falecer no posto de capitão-tenente (Companhia..., 19 ago. 1907).

Nascido em Coimbra, a 20 de setembro de 1865, João Jardim é oriundo de uma família numerosa que se ramifica entre a Figueira da Foz e a sua cidade natal (Falcão, 1987, p.94). Entre os anos letivos de 1883-1884 e 1884-1885, frequentou algumas cadeiras na Faculdade de filosofia da Universidade de Coimbra (Universidade..., 24 mar. 1884, 17 mar. 1885), da qual o seu tio, Manuel Jardim (1818-1887), fora lente e diretor (Falcão, 1987, p.24-25). A sua careira militar começa aos 20 anos de idade, em 1885 (Portugal, 1880-1902). Em 1888 realiza a sua primeira viagem às colónias, encontrando-se na lista de oficiais da estação naval de Macau como “guarda marinha” (Estação..., 23 nov. 1888). João Jardim é promovido a segundo-tenente da armada no ano de 1890 (Ministério..., 7 jun. 1890), continuando a sua carreira em várias colónias portuguesas, com destaque para Macau e Moçambique (Mano, 1997, p.107).

Nomeado em diversas ocasiões para comissões nos territórios coloniais, Jardim não deixou de desenvolver uma relação profissional com a cidade da Figueira da Foz. Foi nomeado capitão do porto por duas vezes, em 1898 e 1900 (Portugal, 1885-1906), tendo por essa altura integrado a Sociedade Arqueológica Santos Rocha, fundada em 1898^4^ (Ferreira, Cardoso, 1999, p.123). Essa sociedade criada por António dos Santos Rocha (1853-1910), também fundador do Museu Municipal, tinha precisamente como objetivo auxiliar a instituição museológica no incremento da produção de conhecimento e no crescimento das suas coleções (SASR, 1904, p.3). É com esse propósito que, entre 1900 e 1903, João Jardim ofereceu a essa sociedade a mencionada coleção de 260 objetos. Destes, 66 são provenientes de Angola, e 194 de Timor-Leste.

Ainda antes de darem entrada no museu, os objetos timorenses são destacados no periódico local *Gazeta Figueira*: “Deu já entrada no Museu Municipal d’esta cidade, onde se está instalando a interessante coleção etnográfica de Timor-Leste, inteligentemente organizada para a Sociedade Arqueológica da Figueira, pelo sócio correspondente sr. João Jardim, ilustre oficial da nossa armada, e oferecida pelo mesmo à referida sociedade” (Museu..., 28 mar. 1900, p.1). Esse periódico descreve também a dimensão e as características da coleção, incluindo as designações dos objetos na “língua Macaçai”, inscritas num documento providenciado à instituição, provavelmente pelo próprio militar. Entre os artefatos coligidos encontravam-se cerâmicas de Baucau, Vémasse e Viqueque, os três reinos sob o domínio português que, de acordo com João Jardim, possuíam essa indústria desenvolvida (Jardim, 1899-1903a, p.823).

Essa coleção doada por João Jardim, incorporada no Museu Municipal a 23 de abril de 1900, seria a mais variada da seção de etnografia e a que possuía documentação mais detalhada, nomeadamente por meio das “Notas etnográficas sobre os povos de Timor”, posteriormente publicada na revista *Portugália* (Jardim, 1899-1903b) e também divulgada na segunda sessão plenária da Sociedade Arqueológica, a 24 de outubro de 1898. As suas descrições, como as de outros atores da época (Roque, 2022, p.315), refletiam as hierarquizações sobre o valor humano:

O timorense é, como todo o selvagem, imprevidente e preguiçoso. Incapaz de qualquer energia cerebral ou muscular, sóbrio em extremo, intensamente voluptuoso, e desconhecendo o bem-estar e conforto da habitação, que o clima quase torna inútil, despreza o trabalho, que lhe não aumenta os gozos, e dormita lazaronicamente ao sol, mascando a areca e o betel, ou sorvendo, a pequenos goles o bambu de tuaca (suco de palmeira) vivificante (Jardim, 1899-1903b, p.357).

Também um estudo sobre cerâmica timorense é apresentado por João Jardim na Sociedade Arqueológica no dia 7 de janeiro de 1900, o qual também veio a ser publicado na revista *Portugália* com o título “A cerâmica em Timor” (Jardim, 1899-1903a).

Os 194 objetos provenientes dessa mesma região foram descritos no livro de Registo das Entradas por Depósito nos seguintes termos:

Vários tecidos, como panos, saias, coletes, sacos; vários artefatos de palha, tais como caixas, tampas de vasos, cestos, cigarreiras; varias peças de cerâmica, umas grosseiras, outras mais finas, ornamentadas; colheres de concha, coco e prata; máquinas de escaroçar algodão; rocas; novelos; sacos e chapéus de palha, tudo proveniente de Timor … varios instrumentos musicais; escudos e cartucheiras de pele de búfalo; bolsas de pele; facas e espadas; lanças de ferro e de bambu; penachos; facha; abanos de palha; batuques; copo de côco; cabaças; manilas de conchas e de prata; pentes para homem e mulher; amostras de barro e graes para moer as tintas, tudo proveniente de Timor... (MMF, 1893, p.85-86).

O militar descreveu também as formas de organização dos reinos e a caracterização dos timorenses, além da descrição do trabalho de cerâmica (Jardim, 1899-1903a; Jardim, 1899-1903b).

No período em que foi recolhida essa coleção, no final do século XIX, ocorriam em Timor-Leste várias campanhas militares procurando submeter as hierarquias tradicionais do território à administração portuguesa (Tomás, 1976, p.498) e também conter as incursões dos holandeses a leste da fronteira. Pélissier (2007, p.263-270) descreve que o então governador, Celestino da Silva, fazia por impor com violência a soberania portuguesa.

A coleção doada por João Jardim foi integrada na “Sala da Comparação”, que apresentava uma seção de etnografia onde se coligiam os objetos “dos povos selvagens dos tempos modernos, compreendendo os objetos das suas artes ou industriais, que possam interessar ao estudo etnográfico dos selvagens dos tempos pré-históricos na Europa” (Rocha, 1905, p.10). Essa organização promovida pelo diretor Santos Rocha transmitia uma clara subalternização dos grupos humanos e sistemas culturais sob o olhar hierarquizante do europeu. Os timorenses, embora classificados num estádio de desenvolvimento que incluía o uso dos metais e de armas de fogo, eram associados a uma mentalidade considerada “primitiva”:

Sem dúvida o indigena de Timor não é hoje, sob muitos pontos de vista, um selvagem como os dos tempos neolíticos na Europa. Ele usa dos metais e até de armas de fogo; está sujeito às leis portuguesas, e recebe o ensino das missões e os artefatos europeus. Mas as indicações dadas pelo sr. Jardim provam que ele conserva no seu modo de pensar e nos seus usos e costumes muito da sua simplicidade primitiva: é ainda um selvagem, embora adornados com atavios da nossa civilização (Rocha, 1899-1903, p.355).

Os estudos comparativos sobre a coleção de Timor-Leste parecem não se concretizar, em parte porque a índole comparativa da seção etnográfica acaba por desaparecer nos anos seguintes. O falecimento de António dos Santos Rocha, em 1910, significou a estagnação das coleções da seção de etnografia e dos seus estudos. Em 1945, com a reabertura do museu sob a direção de Vítor Guerra, a Sala de Comparação passa a designar-se Sala de Etnografia (Pereira, 1986, p.29). Algumas décadas depois, em 1975, os objetos etnográficos, como todo o conteúdo do museu, são transferidos para um novo edifício construído com o apoio da Fundação Calouste Gulbenkian, onde ainda permanecem atualmente. A coleção de Timor-Leste não foi inicialmente exposta na Sala de Etnografia desse novo espaço aberto ao público em 1981 (Pereira, 1982). Ganharia destaque anos depois, em 1994, por meio da criação de uma seção dedicada às “Coleções Orientais” no âmbito da comemoração do centenário do museu (Roteiro..., 1995).

Parte dos objetos oferecidos por João Jardim viria também a assumir projeção nacional e internacional. Em 1988, o “Sasando” (MMSR, 2024b), proveniente de Timor-Leste e então erradamente identificado como proveniente de Madagascar e designado como “Marouwane” (Portugal, 1988, p.46), foi integrado na exposição “África e Índico com Bartolomeu Dias: o encontro para o melhor conhecimento do homem e do instrumental africano”, organizada pelo Instituto Português do Património Cultural na Torre de Belém. Aquando da sua entrada no museu, no ano de 1900, esse objeto surge identificado como “Sandou”, designado por João Jardim como um “instrumento musical grande” (Coleção..., 1900). No *Catálogo geral* de 1905 surge registado com o n.7216 e descrito como “Violão feito d’uma folha de palha” (Rocha, 1905, р.99). Apesar disso, é a designação de “Marouwane” que é replicada num catálogo publicado pelo Museu Municipal no ano seguinte, que o integra numa secção sobre instrumentos musicais africanos (Ávila, 1989). Em 1994 foi alterada a sua designação para “Cordofone, de palma. Séc. XIX”, sendo integrado nas coleções orientais com indicação de que era proveniente de Timor-Leste (Roteiro..., 1995). É nesse contexto que o objeto é pela primeira vez atribuído a João Jardim pela conservadora Isabel Pereira (1995, p.161), já se considerando, portanto, a documentação histórica do museu associada à coleção. Alguns anos mais tarde, surge registado no catálogo comemorativo do centenário da Sociedade Arqueológica com a designação de “Cordofone-Sasando” (Ferreira, Cardoso, 1999, p.208). Designação essa que o acompanha até ao presente.

Uma parte da coleção doada por João Jardim em Timor-Leste foi também requisitada para integrar a exposição internacional que ocorreu em Lisboa em 1998 (“Expo 98”) (Relatório..., 1998), assim como, dois anos depois, a exposição “Cerâmicas de Timor Loro Sa’e” foi apresentada no Centro Científico e Cultural de Macau, também em Lisboa (Gomes, 2001). Nesse mesmo ano, a coleção foi divulgada na revista *Oriente*: “Objetos de Timor Lorosae no Museu Municipal Santos Rocha” (Cardoso, 2004). A projeção nacional e internacional da coleção seguramente terá contribuído para que ela adquirisse um lugar de relevo na exposição permanente do museu municipal aquando da remodelação da Sala de Etnografia em 2014, não incluindo, contudo, informações associadas ao seu contexto de aquisição e às redes que conduziram os objetos até ao museu. Nesse espaço expositivo, que permaneceu inalterado até ao momento presente, um texto de parede introdutório informa o público que a coleção etnográfica do museu tem origem na “seção comparativa” criada pelo fundador, Santos Rocha, e no seu interesse no estudo dos “selvagens do presente”. Não apresenta, porém, nenhuma reflexão crítica sobre essas categorizações com origem no século XIX.

Parte dos artefatos provenientes de Timor-Leste encontram-se em exposição junto a uma coleção proveniente de Angola, onde é possível encontrar o “Deus da Guerra”/“Nkisi” (MMSR, 2024a), também obtido por João Jardim e cujo percurso, que analisaremos seguidamente, revela outros contrassensos e ambiguidades.

## Campanhas militares, a coleção de Angola e o caso do “Deus da Guerra”

A primeira das três figuras que se apresentam atualmente na vitrine central da Sala de Etnografia do Museu Municipal, e que se distingue pelos seus traços antropomórficos, corpo esguio e braços longos, é presentemente acompanhada da seguinte legenda: “Angola. Finais do século XIX. Escultura nkisi”.

Foi obtida por João Jardim durante uma expedição na região dos “Quincunguilas”, enquanto cumpria funções de administrador colonial no norte de Angola no início do século XX. A “Expedição à Quincunguila, na circunscrição do Ambrizete”, assim designada e descrita pelo próprio, realizou-se entre janeiro e março de 1902 (Jardim, 1903a). João Jardim comandou uma coluna militar que terá partido de um porto comercial localizado no litoral norte de Angola (Ambrizete) com destino ao interior, à região dos “Quincunguilas” (Jardim, 1903a, p.252). O objetivo era implantar a soberania portuguesa naquela região e abrir os caminhos comerciais da Quibabuge e da Bamba (p.253).

René Pélissier identifica a “Expedição à Quincuguila”, levada a cabo por João Jardim em 1902, como a quarta expedição contra os Solongo. O autor apresenta João Jardim como um “govenador energético, mas pretensioso” (Pélissier, 2013, p.274), mencionando que aquela missão de “pacificação” juntara seiscentos homens contra as gentes do Tomboco que infligiam “o permanente terror a toda a costa do Ambrizete” (p.274).

Jardim atribui a causa da expedição a uma crise comercial causada pela flutuação dos preços e disponibilidade da borracha, que produzia uma instabilidade no escoamento do produto para os mercados a que se destinava (Jardim, 1903b, p.57). A borracha era um produto que se apresentava, na altura, como de importância estratégica para as receitas das exportações coloniais de Portugal (Alexandre, 2004, p.967) e o país encontrava-se dependente do serviço das populações locais que acondicionavam esse produto (Santos, 2013, p.53).

A relação entre os comerciantes portugueses e as populações locais era, segundo Jardim, marcada pela volatilidade do temperamento e pelos interesses dos grupos que controlavam a abertura e o encerramento dos caminhos comerciais e que perpetuavam contínuas “humilhações” e “injúrias” aos comerciantes portugueses daquele porto comercial (Jardim, 1903b, p.257). Para o militar, esse era o resultado da não efetiva ocupação do litoral e da fragilidade que a descontinuada colonização do interior norte de Angola, do “congo português”, representava para os portugueses (p.265).

A prisão de um dos comerciantes e seus carregadores numa viagem ao interior foi, de acordo com João Jardim, o motivo que desencadeou a campanha militar para submissão daqueles grupos à autoridade portuguesa:

A coluna propôs-se vingar imediatamente essa afronta, tornando a vingança por tal forma duravel em seus efeitos, e por tal modo violenta na sua execução, que inibisse o gentio de repetir atos semelhantes, subjugando-o, abatendo-o n’uma attitude humilde e respeitosa. Por isso se não limitou a destruir: edificou. Dominando a região assolada, enegrecida do incêndio de 38 povoações, ou antes, de 41 – porque o povo do Quitamboco era composto de quatro grandes povos separados – ergueu em 12 dias de trabalho interrupto o forte que Sua Majestade El-Rei houve por bem honrar, concedendo-lhe o seu nome (Jardim, 1903c, p.115).

É durante a descrita investida militar a uma das aldeias que um dos oficiais de João Jardim obteve o artefato descrito como “manipanso” e “Deus da Guerra”:

Mas, de repente, n’uma espécie de pequeno lago, deparou-se-lhe um manipanso pintado a vermelho desmaiado e mosqueado a preto e branco, em manchas que vagamente simulavam os olhos que os barcos algarvios usam nas amuras. Por cima do mono flutuava, na ponta d’um bambu, um trapo vermelho: a bandeira de guerra dos quincunguilas. Naturalmente deitou a mão ao manipanso, e atirou a terra a bandeira. N’esse mesmo instante cessou o fogo, e os guerreiros largaram em tão descompassada fuga que não houve pôr-lhes mais a vista em cima. Era, pelo que se vê, o deus da guerra, o feitiço da valentia, a égide protetora nos combates: todo o arrimo, enfim e toda a força dos lutadores. Uma vez que se deixou tocar pelos brancos, sem responder ao sacrilégio pela fulminação imediata das mãos ímpias, perdeu a virtude, renegando a sua gente. Daí o pânico e a mudança da situação, que os próprios Campos Andrade e Valle me declararam que se ia tornando difícil, por causa do esgotamento das munições. O jovem oficial – que felicitei calorosamente pela sua corajosa atitude, sangue frio e decisão – ficou com a bandeira de guerra, tão justamente ganha, e ofereceu-me o manipanso. É uma escultura quase informe de 1m30 de altura, de crânio pontiagudo, de braços longos, afilada e esguia como uma figura bizantina (Jardim, 1904, p.29, 30).

João Jardim produz observações que admitem um entender estratégico da função daquele objeto nos desfechos armados com os locais. De fato, encontramos nas descrições de outros militares da época o mesmo tipo de entendimento. Em 1900, numa ação militar que se realizou entre São Salvador e o Cuilo, o general José Heliodoro Corte-Real de Faria Leal (1862-1945) descreveu: “Em quase todos os povos existem feiticeiros de guerra que vão dançando em frente dos contendores, afugentando as balas com um penacho de cauda de boi; morto o feiticeiro principal apodera-se quase sempre o pânico dos que o perderam, que quase sempre, se dispersam e fogem” (Leal, 1902, p.258).

Ao longo do século XIX, artefatos como o obtido por João Jardim eram muitas vezes associados às designações pejorativas de “feitiço”, “manipanso” ou “ídolo” (Bastin, 1994b, p.11). Jardim atribuiu-lhe a designação de “Deus da Guerra”, fruto da circunstância em que vê o artefato em utilização. É precisamente sob esse nome que é incorporado no museu da Figueira da Foz e registado no *Catálogo geral* em 1905: “N° 8000 – Estátua itifálica do Deus da Guerra, tomada na povoação de Fardiabengo, região de Quincunguila, por ocasião da guerra do Quitamboco, em março de 1902” (Rocha, 1905, p.109). Como reflete a anterior descrição, os europeus projetavam sobre esses itens tipologias e sistemas de crenças análogos aos seus (Vanhee, 2000, p.89), o que promoveu descrições inadequadas do que seria a sua função e o seu significado no contexto cultural de origem (MacGaffey, 1988, p.190).

O percurso dessa “Nkisi”, atualmente identificada no museu Santos Rocha como “escultura” e “figura de poder” (MMSR, 2024a), mostra como foi objetificada e categorizada. “Nkisi” é uma palavra kikongo da África Central que se pode traduzir por “espírito” para uma maior aproximação ao seu real significado (MacGaffey, 1988, p.191). Nesse contexto, “Nkisi” é entendida como um veículo que é habitado por personalidades do mundo dos mortos, tornando nesse processo acessíveis os seus poderes aos vivos (p.191). Algumas participavam na regulação dos conflitos do quotidiano, assumindo um papel central na vida das comunidades (MacGaffey, 2000, p.64). “Nkisi” não é objeto, mas uma entidade ou um sujeito. Assim, o que encontramos nos museus é apenas um vestígio material de um denso e complexo mundo que deixa de existir quando “Nkisi” é retirada do seu local de origem.^5^

No museu da Figueira da Foz, essa “Nkisi” integra a mencionada coleção de 66 objetos provenientes de Angola oferecidos por João Jardim, correspondendo o período da sua obtenção à época em que este ator esteve na ex-colónia portuguesa. Nessa coleção, que deu entrada no museu a 28 de janeiro de 1903, registam-se “varios ídolos de madeira, esculturas da mesma matéria, espadas, artefatos de palha, artefatos de madeiras, um fole, um fuso e respectiva roca, amostras de (?) e tacula, manilhas de bronze, ídolos de osso; escultura de marfim, tambores de festa e de guerra, um capacete de penas, tudo prov. do congo portuguez” (MMF, 1893, p.96). Esse conjunto reúne uma série de objetos que se enquadram na categoria de “Nkisi”, constituindo a maior entrada de itens dessa tipologia no museu. Alguns são descritos pelo fundador como “Amuletos”, outros como “Mabiala (ídolo)” (Rocha, 1905, p.108).

A “Nkisi”, a quem o militar chamou “Deus da Guerra”, esteve permanentemente em exposição, integrando as sucessivas reestruturações e reinterpretações da coleção de etnografia. Em 1903, aquando da sua integração na Sala de Comparação, representava a cultura material dos “primitivos contemporâneos”, e, extinto o espaço de comparação, passou para a Sala de Etnografia. Em 1976, integrou a exposição itinerante “Angola. Culturas tradicionais”, organizada pelo Instituto de Antropologia da Universidade de Coimbra com o objetivo de dar a “conhecer também as variações das características culturais, permitindo ilustrar os modos de vida das diferentes etnias” (Figueiras et al., 1976, p.15). Nesse contexto foi atribuída aos Bakongo, a quem são associados objetos tipo “estatuetas-relicário, possivelmente de inspiração cristã; os característicos ídolos cravejados de pregos, de significado algo misterioso, ligados sem dúvida às práticas de feitiçaria” (p.58). Nesse mesmo catálogo é descrita como “Estatueta hermafrodita. Que se integra no estilo geral dos Bakongo pelas características do rosto e do penteado” (p.69).

No Museu Municipal, em 1981, a “Nkisi” vai integrar a “Coleção do norte de Angola” na exposição da Sala de Etnografia, onde é identificada como “Estatueta relicário, hermafrodita” (Pereira, 1982). Em 1995, é retomada a interpretação de João Jardim, surgindo identificada como “Figura humana representando o Deus da Guerra” (Roteiro..., 1995). Nesse ano também integrará a exposição do Museu Nacional de Etnologia, “Escultura angolana. Memorial de culturas”, organizada por Marie Louise Bastin. No respectivo catálogo é atribuída aos Solongo do Tomboco e novamente identificada como “Deus da Guerra”: “Excepcional representação longiforme curiosamente hermafrodita, o ‘Deus da guerra’, erguido altivamente, apoiando as mãos sobre a parte inferior do abdómen e com a ‘carga’ resinosa na parte superior. Ritualmente realçada de preto na face, e de vermelho tracejado de branco, no corpo” (Bastin, 1994b, p.76). A mesma exposição será também apresentada na Bélgica, no Stad Antwerpen Etnografich Museum (Bastin, 1994a). Uma década volvida, em 2005, essa designação é novamente apresentada em Madrid, na exposição “Orígenes. Artes primeras. Colecciones de la Península Ibérica. América, Oceanía, Asia e África” (Gaspar et al., 2006).

Na sua trajetória dentro do museu, a designação “Nkisi” surge em cerca de 2010, depois de ter sido exibida em França na exposição “Angole, figures de pouvoir” no Musée Dapper (Falgayretter-Leveau, 2010, p.34). Paralelamente, desde 2014 que o Museu Municipal Santos Rocha, com a transformação da Sala de Etnografia nesse ano, a apresenta com a legenda “Angola. Finais do século XIX. Escultura nkisi”.

## Considerações finais

Ao revelarem dimensões que ultrapassam a sua própria materialidade, e nos informam sobre as leis e regulamentos da vida social e cultural que acompanham e representam, os objetos dos museus podem ser considerados como atores ou agentes da história (Hoskins, 2006, p.82). Seguir os significados que vão adquirindo ao longo das suas vidas, nomeadamente dentro dos museus, implica identificar a rede de relações sociais na qual eles se integram ou integraram (Foster, 2012). A exploração das redes dos objetos, que envolvem pessoas e coleções, mostra-nos a ação daqueles na esfera social da vida humana, ou seja, como objetos e pessoas estão interligados e mutuamente se definem em valores e significados (Foster, 2012, p.152). A pesquisa dessas redes descodifica relações frequentemente invisíveis na narrativa expositiva dos museus, apelando a conexões mais profundas entre objetos-pessoas e momentos da nossa história comum (Pereira, Carvalho, Cardoso, 2021).

Em alguns casos, traçar a origem dos objetos revela-se um processo complexo. Apesar de identificados os doadores, as pistas sobre a sua proveniência podem dissipar-se por carência de fontes. É frequente a ausência de informação sobre os processos de aquisição, sobre os coletores ou colecionadores, o momento de recolha, obtenção ou compra, sobre os intricados encontros e narrativas históricas associadas, e também sobre as identidades das populações sujeitas ao colonialismo a quem pertenciam os bens culturais. Os objetos que destacamos neste artigo, ainda que indissociáveis de regiões geográficas e de um doador identificado e registado na documentação do museu, foram erradamente interpretados durante o seu itinerário na instituição e fora dela. Exemplificativo é o “Sasando”, instrumento musical obtido em Timor-Leste por João Jardim, o qual foi apresentado na década de 1980 como proveniente do continente africano. Nesse caso mostra-se a necessidade de conhecer o historial das coleções, uma vez que prevaleceram nos museus as visões eurocêntricas que as apresentam somente como curiosidades representativas de uma alteridade.

Por outro lado, a “Nkisi” obtida em Angola tem sido, durante décadas, entendida e designada com a terminologia colonial de “Deus da Guerra”, o que evidencia como a documentação dos museus tende a registar informação filtrada, que apresenta fragmentos da realidade. Por esse motivo, é crucial criticar as fontes, indo além da documentação dos museus para identificar a colonialidade das biografias de uma grande parte dos doadores e dos momentos sócio-históricos evocados. Associar os registos dos museus com outros materiais de arquivo proporciona uma visão mais ampla do colonialismo e dos seus efeitos nas periferias, seja nas colónias ou nas metrópoles.

A existência de uma coleção comparativa na Figueira da Foz, com objetos coloniais, evidencia como o mundo era ordenado, classificado e apresentado ao público. Os museus e as coleções etnográficas não espelhavam a realidade, criavam uma nova realidade, paralela à legitimação dos agentes coloniais. No caso de João Jardim, podemos encontrar nas “Portarias provinciais” do Governo Geral de Angola de 18 de junho de 1902 um louvor aos “oficiais da Armada pelas operações na região das Quincunguilas, no Ambrizete, em fevereiro e março de 1902” (Portarias..., 2 set. 1902, p.2717). Essa campanha resultou no incêndio e na destruição de 41 aldeias, e consequente dominação da região do Quitamboco, na construção do forte com a designação “D. Carlos I” (Jardim, 1903c, p.115) e na doação de uma coleção ao Museu Municipal Santos Rocha.

As coleções coloniais apresentam-se, assim, como formas de negociação de poder nas quais transparecem princípios de conhecimento ocidentais. Nos museus, essas configurações manifestam-se em vários processos de significação e ocultação, que se identificam, por exemplo, no apagamento das redes de atores que fazem parte da construção das coleções. A documentação do percurso dessas coleções levanta uma série de questões sobre o que está a ser representado nesses objetos e sobre o que não está a ser contado das suas histórias, para as quais não encontramos respostas nas narrativas do museu.

A disponibilidade dos museus portugueses em criar espaço para pesquisas de proveniência é fundamental para promover transparência sobre os processos de construção das coleções coloniais em Portugal. Uma realidade que tem enfrentado inúmeros desafios e dificuldades, pelo que destacamos o assinalável investimento do Museu Municipal Santos Rocha na promoção de investigação sobre as suas coleções.

**Figura 1:**
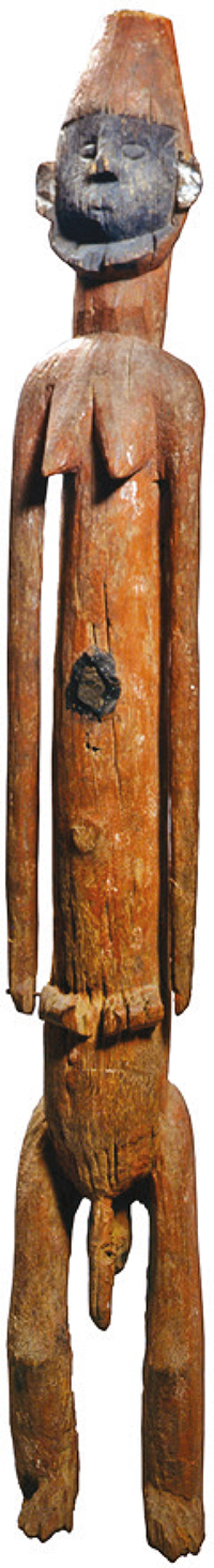
: “Nkisi”, foto de Delfim Ferreira, ano incerto (MMSR, 2024a)

## NOTAS

1 Como exceção, destaca-se a tese de mestrado de Liliana Caldeira (2023), que desenvolve uma estratégia semelhante à apresentada neste artigo para a abordagem de objetos das coleções do Museu Nacional de Arqueologia, por meio da matriz do projeto TRANSMAT.

2 É, por exemplo, o caso da Bélgica, no African Museum (Van Beurden, Gondola, Lacaille, 2023), e da Alemanha, no estudo das coleções do antigo Ethnologishes Museum, atualmente no Humbolt Forum (Von Oswald, 2022).

3 No âmbito deste projeto foi desenvolvida uma base de dados para análise das coleções do Museu Municipal Santos Rocha e Museu Nacional de Arqueologia: TRANSMAT – Transnational materialities (1850-1930): reconstituting collections and connecting histories (PTDC/FER-HFC/2793/2020).

4 Inicialmente designada Sociedade Arqueológica da Figueira, torna-se Sociedade Arqueológica Santos Rocha por decisão da sessão plenária de 1903 (SASR, 1904, p.4).

5 Estes objetos encontram-se em vários museus europeus (African Museum, Bruxelas; Humbolt Fórum, Berlim; Quain Branly, Paris; Brittish Museum, Londres; Pitt Rivers, Oxord etc.). Em Portugal, no Centro Internacional das Artes José de Guimarães, no espólio do extinto Museu de Antropologia da Universidade de Coimbra, no Museu Nacional de Etnologia, na Sociedade de Geografia de Lisboa e no Museu Municipal de Lagos.
